# Comment on ‘Treatment of idiopathic granulomatous mastitis and factors related with disease recurrence’

**DOI:** 10.3906/sag-2006-80

**Published:** 2020-12-17

**Authors:** Emre TEKGÖZ, Seda ÇOLAK, Muhammet ÇINAR, Sedat YILMAZ

**Affiliations:** 1 Department of Internal Medicine, Division of Rheumatology, Gülhane Faculty of Medicine, University of Health Sciences, Ankara Turkey

Dear Editor,

First of all, we would like to thank the authors for the criticism of and contributions to our recent article about idiopathic granulomatous mastitis (IGM) [1]. IGM is a rare disease; however, arousing interest in it is seen in recent years.

In the current study, our IGM treatment experience was presented from a rheumatology perspective. The patients, who had a histopathological appearance of granulomatous inflammation in breast specimens, were referred to our rheumatology outpatient clinic for differential diagnosis of inflammatory disease of the breast. The patients who participated in our study were referred to our clinic from general surgery clinics of our hospital or other centers. All the patients had already been treated with antibiotics or surgical interventions, such as drainage, at the referring centers. These procedures are not the routine practice of our rheumatology clinic. The unresponsive patients were referred to our clinic and were reevaluated primarily for infectious and noninfectious causes of granulomatous diseases. To us, it is more rational to make the differential diagnosis by a rheumatologist since the other granulomatous diseases mimicking IGM are somewhat familiar to the rheumatology practice. Making the differential diagnosis is more complicated than ordering “autoimmune markers”. We have diagnosed one case of eosinophilic granulomatosis with polyangiitis, two cases of sarcoidosis, and one case of tuberculosis in the patients referred to us as IGM. After excluding these diseases, the patients were followed up with the diagnosis of IGM in our rheumatology outpatient clinic.

There is no consensus on the treatment algorithm for IGM. Antibiotics, surgical intervention, glucocorticoids (GCs), and immunosuppressive therapy are options. In the current study, we reported our experience, especially about methotrexate (MTX). In the literature, the recurrence rate of IGM has been reported as high as 50%. Therefore, it is obvious that the need for alternative approaches is mandatory. Although follow-up period is relatively short in our study, the recurrence rate is quite low in comparison to the reported literature data. Based on our MTX experience, which is incomparable to the surgeons, we initiate MTX for almost all IGM patients concurrently with GCs. Although MTX is also known as an antineoplastic agent and has potential side effects, it is quite safe in the long term in doses used in rheumatology practice with appropriate precautions. MTX is used and recommended to decrease or prevent the serious adverse effects of GCs in patients with other inflammatory diseases, which constitutes our rationale behind initiating this agent as early as possible [2,3]. Besides, the initiating of MTX as low as 5 mg/week is quite far from its therapeutic dosage. If the patient has no concomitant diseases, such as renal or hepatic disease, MTX should be initiated at a minimum dose of 10 mg/ week. The efficacy of MTX treatment appears after 2–4 weeks of treatment. Appropriate dose titration can be made with an increase of 2.5–5 mg/week every 4–6 weeks. Generally, doses of 10–15 mg weekly are sufficient in most of the patients. Doses may be increased to 25 mg/week [3]. In the case of remission, firstly, GC dose should be reduced and discontinued if possible. There is no standard approach to MTX dose reduction, which can be applied for those who have been in remission for at least 6 months. In our clinical practice, the dose is reduced 2.5 mg every 3 months to the minimum effective dose (7.5 mg/week). The cessation should be considered in patients who have been in remission for at least 6 months with the lowest effective dose of MTX. If relapse occurs during tapering MTX or GC, the dose is increased to the previous effective dose immediately. If remission cannot be achieved, either increasing the dose or switching to another immunosuppressive treatment alone or in combination are options for the treatment.

Classifying IGM as a benign disease only because it does not have malignant features should be taken as controversial. According to this approach, we need to consider other inflammatory disorders, such as rheumatoid arthritis or vasculitis as benign diseases, which should be questioned in terms of the indication of MTX and GCs. IGM may potentially cause cosmetic, physiological, and psychological undesirable results. For this reason, to prevent these unfavourable outcomes, and to protect patients from short and long term adverse events of GC, initiating MTX early with GC seems feasible, which corresponds to our therapeutic approach.

Finally, since there is a growing body of evidence about imaging modalities, such as USG and MRI, that can be used in the diagnosis of IGM, we needed to emphasize the indispensable role of biopsy [4,5].

The rheumatology follow-up of the IGM patients who were presented to our rheumatology outpatient clinic was evaluated in the current study. We extend our thanks to the authors for their interest and allowing us to emphasize our contributions.
